# Dilatation and Curettage Effect on the Endometrial Thickness

**DOI:** 10.5812/ircmj.9863

**Published:** 2013-04-05

**Authors:** Robab Davar, Razieh Dehghani Firouzabadi, Kefayat Chaman Ara

**Affiliations:** 1 Department of Obstetrics and Gynecology, Shahid Sadoughi University of Medical Sciences, Yazd, IR Iran

**Keywords:** Dillatation and Curretage, Endometrial Thickness, Transvaginal Ultrasonography

## Abstract

**Background:**

Endometrial receptivity is required for successful implantation and pregnancy. Despite the remaining controversy, many studies have shown that ultrasonographic endometrial thickness can be considered as an indicator of endometrial receptivity.

**Objective:**

The study objective was to investigate the effect of dilatation and curettage on the endometrial thickness.

**Materials and Methods:**

Enrolled in the study were 444 patients visited in Obstetrics & Gynecology clinic of Shahid Sadoughi hospital between Jan. 2011 to Sep. 2012. Only patients whose menstrual cycle was regular were included in study. Patients with myoma, adenomyosis, endometrial polyps or other uterine anomaly, those who smoked, whose BMI was greater than 30 and who were taking medications that could affect endometrial thickness were excluded. Endometrial thickness was measured one day before evolution (n = 444) and 5-7 days after it (n = 444) using transvaginal ultrasonography. The endometrial thicknesses were correlated to the patients’ history of dilatation and curettage. Data analysis was done through SPSS software version 16 and using descriptive statistics, independent T-test and Anova.

**Results:**

Endometrial thickness in patients who had 0, 1, 2, 3 and 4 D&C were 10.00 ± 0.58, 9.83 ± 0.47, 8.90 ± 0.92, 7.42 ± 0.18 and 7.40 ± 0.07, respectively one day before ovulation (spearman’s correlation coefficient = -0.33) and 10.62 ± 0.68, 9.64 ± 0.49, 8.48 ± 0.96, 6.32 ± 0.15 and 6.90 ± 0.04, respectively, 5-7 days after ovulation (spearman’s correlation coefficient = -0.66) estradiol and progesterone levels, measured in the day of 2nd ultrasonography had not statistic relation with endometrial thickness (P = 0.27 and 0.31). The relation of endometrial thickness and age was not significant (P = 0.54 and 0.06).

**Conclusions:**

Dilatation and curettage has a significant effect on the endometrial thinning.

## 1. Background

Human endometrium is a fascinating, dynamic, steroid-responsive tissue that undergoes repeat cycles involving sequential proliferation, differentiation, breakdown and repair (1, 2). These changes are regulated in the presence of estradiol and progesterone (3). Its sole purpose is to enable implantation of an embryo during a relatively short window of opportunity in the menstrual cycle (1). Endometrial receptivity is required for successful implantation in natural and IVF cycles (4, 5). There are still no accepted criteria for evaluating endometrial receptivity (5) but as endometrial morphology may reveal its readiness, endometrial morphologic features have all been evaluated as markers of receptivity and consequently implantation and pregnancy. (6). Endometrial thickness is one of these features that has been utilized as an indirect indicator for endometrial receptivity (7). Endometrial thickness has been said to affect the successful outcome pregnancy (8). Numerous studies have focused on determining uterine receptivity through sonographic evaluation of the endometrial thickness but have been unable to reach a consensus (9). Some investigators have demonstrated a positive correlation between endometrial thickness and pregnancy rates, (3, 7, 10-21) while others have not found such an association (22-28), or only in association with other parameters (4, 29).

Researchers who believe this correlation exists acknowledge that there are conflicting findings as to the minimum thickness required to support a pregnancy (9). The endometrial thickness found to correlate with a positive pregnancy outcome varies between 6 mm and 10 mm, although studies have reported pregnancies with a thickness of as little as 4 mm. (3, 8, 11-13, 15, 16, 19, 30-33) Also, data in the literature are scant regarding the maximum endometrial thickness that will correlate with pregnancy rates and implantation rates (9). Various investigators have been reported the maximum endometrial thickness of 12 to 16 mm that allows the successful implantation and pregnancy, although studies have been reported pregnancies with thicknesses of 19 and 20 mm (9, 12, 27, 31, 34). However, all these differences may be in part attributed to different patient populations, stimulation protocols used or measurement timing (5, 9). But, even studies suggesting that there is no correlation between endometrial thickness and pregnancy and implantation rates have reported that no pregnancy occurred if the endometrium measured < 7 mm (9). Therefore it is known that abnormally thin endometrium leads to low pregnancy rates and existent absolutely few studies indicate that curettage (D&C) could be a cause of scant endometrial tissue (5, 35). Dilatation and curettage, a blind procedure, is the most frequent surgical procedure performed throughout the world routinely by many gynecologists (36, 37). It can be used as a diagnostic test or as a form of treatment for a range of health conditions affect women. These problems include abnormal menstrual bleeding, Polyps, uterine infection, incomplete abortion, surgical abortion, heavy bleeding after childbirth, investigations of female infertility, benign tumors, malignant cancer or suspicion of uterine cancer, adenomyosis and pelvic inflammatory disease (38). Although, D & C was the traditional gold standard for endometrial evaluation for many decades (39) , and it said that a D&C procedure is very safe, but there are several possible risks and complications. These are very unlikely but possible (38). Some of the possible complications of D&C include risks of general anesthesia and reactions to the medications used, cervical damage due to dilation or the passage of instruments, puncture or perforation of the uterus wall that could potentially lead to injury of other pelvic structures such as the intestines, the bladder or the blood vessels and nerves, hemorrhage, infection of the uterus or other pelvic organs, scar tissue within the uterus if the scraping done too vigorous, synechiae or intrauterine adhesions (Asherman’s syndrome) and adverse future reproductive outcome, uterine fistulae and death (36-38, 40-43). Even in the absence of these complications, the cost, in terms of hospitalization and absenteeism from work, is substantial (37). Although few studies have been reported the role of D&C in the etiology of thin endometrium (5, 35) but the precise relation between D&C and ultrasonographically thin endometrium remains controversial (35).

## 2. Objectives

Therefore, this research was aimed to investigate the effect of D&C on the endometrial thickness.

## 3. Materials and Methods

Enrolled in the study were 444 patients visited in Obstetrics & Gynecology clinic of Shahid Sadoughi hospital between Jan. 2011 to Sep. 2012. Only patients whose menstrual cycle was regular were included in study. Patients with myoma, adenomyosis, endometrial polyps or other uterine anomaly, those who smoked, whose BMI was greater than 30 and who were taking medications that could affect endometrial thickness were excluded. Endometrial thickness (ET) was measured one day before ovulation (n = 444) and 5-7 days after it (n = 444) using transvaginal ultrasonography. Estradiol and progesterone level were measured in the day of 2nd ET measurement. Endometrial thicknesses were correlated to number of previous performed D&C. Also, endometrial thicknesses were compared between categories of age (< 20, 20-30, and ≥ 30 years). Estradiol level was categorized to 3 categories (< 44, 44-196 and ≥ 196 pg/ ml). Progesterone level also was categorized to 3 categories (< 2, 2-25 and > 25 mg/ml). Endometrial thicknesses were compared between categories of estradiol and progesterone levels. All data were collected through designed forms. Data analysis was done through SPSS software version 16 and using descriptive statistics, independent T-test and ANOVA.

## 4. Results

A total of 444 patients participated in the study. Endometrial thicknesses were measured as 9.80 ± 0.80 and 10.13 ± 1.17 mm, one day before ovulation and 5-7 days after it. 5% (n = 20) and 4% (n = 19) of patients had a thin endometrium (< 7 mm) in two measurements, respectively. Only, 0.9% (n = 4) patients had a history of infertility (Table 1).

**Table 1.** Descriptive Statistics of Research Sample

Variable	No.	Minimum	Maximum	Mean ± SD
**Age, y**	437	18.00	39.00	27.42 ± 4.45
**Gravidity**	444	1.00	7.00	2.13 ± 1.20
**Parity**	439	0.00	5.00	1.34 ± 1.11
**BMI**	423	23.00	25.20	24.34 ± 0.56
**Endometrial thickness (one day before ovulation, mm)**	444	7.20	11.70	9.80 ± 0.80
**Endometrial thickness (5-7 days after ovulation, mm)**	444	6.10	12.80	10.13 ± 1.17
**Estradiol, pg/m**	412	23.90	415.00	169.40 ± 64.05
**Progesterone , mg/ml**	419	0.20	50.00	7.31 ± 6.90

Endometrial thicknesses in patients with D&C history were 10.00 ± 0.58 and 10.62 ± 0.68 mm in two measurements, respectively. Patients with history of D&C had thinner endometrium in booth measurements. The differences between endometrial thicknesses of two groups (with and without D&C history), were statistically meaningful in booth measurements. Those, without D&C history had thicker endometrium, one day and 5-7 days after ovulation (Table 2).

**Table 2.** Mean ETS in Each Groupof D&C Incidence

D & C History		ET (1st measurement), Mean ± SD	Significant	ET (2nd measurement), Mean ± SD	Significant^a^
**With D & C history, No. (%)**		9.33 ± 1.02	0.000^a^	8.98 ± 1.28	0.000
1	92 (20.70)				
2	20 (4.50)				
3	16 (3.60)				
4	4 (0.90)				
Total	132 (20.70)				
**Without D & C history, No. (%)**	312 (70.30)	10.00 ± 0.58		10.62 ± 0.68	

^a^Sig at P < 0.01

We correlated the mean endometrium thicknesses with number of previous performed D&C. Spearmen’s correlation coefficient were -0.33 AND -0.66 for two measurements, respectively. This finding shows a negative correlation between number of D&C and endometrial thicknesses (Table 3).

**Table 3.** Correlation of ETswith Number of Previous Performed D & C

D&C Number	0	1	2	3	4	Significant^a^	Spearman’s Correlation coefficient
**ETs, Mean ± SD**							
1st measurement	10.00 ± 0.58	9.83 ± 0.47	8.90 ± 0.92	7.42 ± 0.18	7.40 ± 0.07	0.000	-0.33^b^
2nd measurement	10.62 ± 0.68	9.64 ± 0.49	8.48 ± 0.96	6.32 ± 0.15	6.90 ± 0.04	0.000	-0.66^b^

^a^Sig. at P < 0.01

^b^Correlation is significant at the 0.01 level (2-tailed)

Endometrial thickness of 5-7 days after ovulation in patients with estradiol levels of < 44, 44-196, ≥ 196 pg/ml were 10.40 ± 0.06, 10.07 ± 1.25 and 10.34 ± 0.59, respectively. Endometrial thickness of 2nd measurement in patients with progesterone levels of < 2, 2-25, ≥ 25 mg/ml were 10.55 ± 0.23, 10.09 ± 1.21 and 10.02 ± 0.42, respectively. Endometrial thickness of 5-7 days after ovulation had not a statistical difference between patients with different estradiol and progesterone level (P value = 0.27 and 0.31) (Table 4).

**Table 4.** ETs Differences Basedon Estradiol and Progesterone Level

	Estradiol Level			Progesterone Level		
	Sum of squares	Mean square	P value	Sum of squares	Mean square	P value
**Endometrial thickness (5-7 days after ovulation)**	3.69	1.84	0.27	3.28	1.64	0.31

Endometrial thicknesses in booth measurements showed an increasing when patient’s age were increased from < 20 to 20-30 years but decreased twice in age group of ≥ 30 but ANOVA test did not showed a statistical difference between endometrial thickness and age in booth measurements. (P value = 0.54 and 0.06) (Table 5).

**Table 5.** ETS Differences Basedon Age Group

Age group, y	< 20 (n = 13)	20-30 (n = 288)	> 30 (n = 136)	P value
**ETs, mm, Mean ± SD**				
ETs (1st measurement)	9.55±1.22	9.81±74	9.76±90	0.54
ETs (2nd measurement)	9.50±1.49	10.21±1.10	9.99±1.31	0.06

## 5. Discussion

Transvaginal ultrasonography (TVUS) is a noninvasive and relatively inexpensive diagnostic procedure to detect endometrial pathology(44). As a result of its low procedure risk, lack of complications and high patient acceptance, and due to limited resources and need to limit costs, transvaginal ultrasound has gained increased worth, recently (45). TVUS can provide serial information about the characteristics of the endometrium (32). It can reliably measure ETh (46). Transvaginal ultrasound with measurement of endometrial thickness can be used to discriminate between normal and pathological endometrium (47). In this study we used TVUS for measuring ETs in a sample of 444 patients visited in our department. The mean age of our sample was 27.42 ± 4.45. ETH was 9.80 ± 0.80 and 10.13 ± 1.17 mm, one day before ovulation and 5-7 days after it. If we use 7mm as the cut of limit for distinguishing between thin and normal endometrium, about 5% and 4% of our sample had thin endometrium, in 2 measurements. Various investigators have been reported that a minimum endometrial thickness (> 7mm) is required for successful pregnancy (9). The incidence of thin endometrium in natural cycles had been reported to be 5% in women < 40 years of age and 25% in 41 to 45 years old women (5). Our sample, all had < 40 years of age., we cannot have any conclusion from this finding. Also, some studies have been reported the detrimental effect of increased endometrial thickness (> 14 mm) on the pregnancy rates. In this study we had not the same thickened endometrial. In our patients the maximum endometrial thicknesses were 11.70 and 12.80 mm in one day and 5-7 days after ovulation, respectively. Among our patients sample a total of 132 patients had the history of dilatation and curettage. They had a total of 199 D&C, 172 for incomplete abortion, 16 for ectopic pregnancy and 11 for hydatidiform mole. Endometrial thicknesses in patients with and without D&C history in first and second measurement were 9.33 ± 1.02, 10.00 ± 0.58 and 8.98 ± 1.28, 10.62 ± 0.68. This findings show that endometrial thickness in each time of cycle were lower in patients with D&C history. Independent sample T-test confirmed these differences where P value was < 01 in booth measurements. Endometrial thicknesses in patients who had 0, 1, 2, 3 and 4 D&C were 10.00 ± 0.58, 9.83 ± 0.47, 8.90 ± 0.92, 7.42 ± 0.18 and 7.40 ± 0.07 in 1st measurement and 10.62 ± 0.68, 9.64 ± 0.49, 8.48 ± 0.96, 6.32 ± 0.15 and 6.90 ± 0.04 in 2nd measurement. Spearman’s rank correlation coefficient of -0.33 and -0.66 indicated a negative relationship between history of D&C and endometrial thickness. Also, endometrial thicknesses of patients with different number of previous D&C were statistically different. (P value = 0.00 and 0.00 for two measurements). Thus the endometrium showed significant thinning after repeated D&C. these finding are in consistent with those of Azomaguchi et al. (2011) (35). They reported the first examination of the relation between endometrial thickness and patient’s history of D&C. In fact we repeated their investigation in our department. Spearman’s correlation coefficients were -0.34 and -0.39 in Azomaguchi et al. study that confirmed with ours. Also, Moon et al. (2009) has been reported the effect of D&C on the subsequent endometrial development in patients undergoing in vitro fertilization (46). Shufaro et al. (2008), in their study have been indicated that thin, unresponsive endometrium could be a possible complication of surgical curettage (5). All these findings strengthen our findings about the relation of endometrial thinning and D&C history. Other findings of our study demonstrated that the serum estradiol and progesterone levels have not a significant relation with endometrial thickness. (P value = 0.27 and 0.31) This findings, also in consistent with those of Azomaguchi et al. (35). The same findings have been reported in some another investigations (48), however some another studies have been indicated that there is a positive relation between serum estradiol concentration and endometrial thickness (14, 19, 28). Our observation regarding the age indicated that there is not a significant relationship between age and endometrial thickness. The study of Azumaguchi et al. has reported a same result regarding the effect of patients’ age on the endometrial thickness. (35). In conclusion, our study revealed the effect of D&C history on the endometrial thinning. Its clinical application could be that the intrauterine invasive procedures such as D&C are devastating to the future endometrial development and reproductive. Therefore, the alternatives should be considered in women of child bearing age. It is notable that, although normally, the endometrium possesses regeneration ability (49) but studies demonstrated that the patients have a poor pregnancy outcome even if some endometrial thickening occur after its’ damage (5). So, avoiding from blind D&C procedure, as much as possible could be suggestible.

## References

[1] Revel A (2012). Defective endometrial receptivity.. Fertil Steril..

[2] King AE, Critchley HO (2010). Oestrogen and progesterone regulation of inflammatory processes in the human endometrium.. J Steroid Biochem Mol Biol..

[3] Kovacs P, Matyas S, Boda K, Kaali SG (2003). The effect of endometrial thickness on IVF/ICSI outcome.. Hum Reprod..

[4] Rinaldi L, Lisi F, Floccari A, Lisi R, Pepe G, Fishel S (1996). Endometrial thickness as a predictor of pregnancy after in-vitro fertilization but not after intracytoplasmic sperm injection.. Hum Reprod..

[5] Shufaro Y, Simon A, Laufer N, Fatum M (2008). Thin unresponsive endometrium--a possible complication of surgical curettage compromising ART outcome.. J Assist Reprod Genet..

[6] Boys E, Chapman MS (2012). Review: The Influence of endometrial thickness on IVF outcome.. J Fert In Vitro..

[7] Al-Ghamdi A, Coskun S, Al-Hassan S, Al-Rejjal R, Awartani K (2008). The correlation between endometrial thickness and outcome of in vitro fertilization and embryo transfer (IVF-ET) outcome.. Reprod Biol Endocrinol..

[8] Bassil S (2001). Changes in endometrial thickness, width, length and pattern in predicting pregnancy outcome during ovarian stimulation in in vitro fertilization.. Ultrasound Obstet Gynecol..

[9] Dietterich C, Check JH, Choe JK, Nazari A, Lurie D (2002). Increased endometrial thickness on the day of human chorionic gonadotropin injection does not adversely affect pregnancy or implantation rates following in vitro fertilization-embryo transfer.. Fertil Steril..

[10] McWilliams GD, Frattarelli JL (2007). Changes in measured endometrial thickness predict in vitro fertilization success.. Fertil Steril..

[11] Chen SL, Wu FR, Luo C, Chen X, Shi XY, Zheng HY (2010). Combined analysis of endometrial thickness and pattern in predicting outcome of in vitro fertilization and embryo transfer: a retrospective cohort study.. Reprod Biol Endocrinol..

[12] Osemwenkha AP, Osaikhuwuomwan JA (2012). Correlation between endometrial thickness and IVF outcome in an African population.. Gynecol Obstet..

[13] Okohue JE, Onuh SO, Ebeigbe P, Shaibu I, Wada I, Ikimalo JI (2009). The effect of endometrial thickness on in vitro fertilization (IVF)-embryo transfer/intracytoplasmic sperm injection (ICSI) outcome.. Afr J Reprod Health..

[14] Remohi J, Ardiles G, Garcia-Velasco JA, Gaitan P, Simon C, Pellicer A (1997). Endometrial thickness and serum oestradiol concentrations as predictors of outcome in oocyte donation.. Hum Reprod..

[15] Noyes N, Liu HC, Sultan K, Schattman G, Rosenwaks Z (1995). Endometrial thickness appears to be a significant factor in embryo implantation in in-vitro fertilization.. Hum Reprod..

[16] Traub ML, Van Arsdale A, Pal L, Jindal S, Santoro N (2009). Endometrial thickness, Caucasian ethnicity, and age predict clinical pregnancy following fresh blastocyst embryo transfer: a retrospective cohort.. Reprod Biol Endocrinol..

[17] Momeni M, Rahbar MH, Kovanci E (2011). A meta-analysis of the relationship between endometrial thickness and outcome of in vitro fertilization cycles.. J Hum Reprod Sci..

[18] Wiweko B, Hestiantoro A, Natadisastra M, Sumapradja K, Mansyur E (2010). Not only embryo quality but also Endometrial Thickness Contributes to IVF outcome: a retrospective study of all IVF cycles in Yasmin Clinic, Jakarta, Indonesia.. Indones J Obstet Gynecol..

[19] Yamashita Y, Hosotani T, Morishima S, Mori K, Ushiroyama T, Ueki M (2003). The Outcome of repeated In vitro fertilization-embryo transfer based on the endometrial thickness.. T Bulletin of the Osaka Medical College..

[20] De Geyter C, Schmitter M, De Geyter M, Nieschlag E, Holzgreve W, Schneider HP (2000). Prospective evaluation of the ultrasound appearance of the endometrium in a cohort of 1,186 infertile women.. Fertil Steril..

[21] Richter KS, Bugge KR, Bromer JG, Levy MJ (2007). Relationship between endometrial thickness and embryo implantation, based on 1,294 cycles of in vitro fertilization with transfer of two blastocyst-stage embryos.. Fertil Steril..

[22] Detti L, Yelian FD, Kruger ML, Diamond MP, Rode A, Mitwally MF (2008). Endometrial thickness is related to miscarriage rate, but not to the estradiol concentration, in cycles down-regulated with gonadotropin-releasing hormone antagonist.. Fertil Steril..

[23] Jarvela IY, Sladkevicius P, Kelly S, Ojha K, Campbell S, Nargund G (2005). Evaluation of endometrial receptivity during in-vitro fertilization using three-dimensional power Doppler ultrasound.. Ultrasound Obstet Gynecol..

[24] Kinay T, Tasci Y, Dilbaz S, Cinar O, Demir B, Haberal A (2010). The relationship between endometrial thickness and pregnancy rates in GnRH antagonist down-regulated ICSI cycles.. Gynecol Endocrinol..

[25] Merce LT, Barco MJ, Bau S, Troyano J (2008). Are endometrial parameters by three-dimensional ultrasound and power Doppler angiography related to in vitro fertilization/embryo transfer outcome?. Fertil Steril..

[26] Puerto B, Creus M, Carmona F, Civico S, Vanrell JA, Balasch J (2003). Ultrasonography as a predictor of embryo implantation after in vitro fertilization: a controlled study.. Fertil Steril..

[27] Rashidi BH, Sadeghi M, Jafarabadi M, Tehrani Nejad ES (2005). Relationships between pregnancy rates following in vitro fertilization or intracytoplasmic sperm injection and endometrial thickness and pattern.. Eur J Obstet Gynecol Reprod Biol..

[28] Zhang X, Chen CH, Confino E, Barnes R, Milad M, Kazer RR (2005). Increased endometrial thickness is associated with improved treatment outcome for selected patients undergoing in vitro fertilization-embryo transfer.. Fertil Steril..

[29] Reuter KL, Cohen S, Furey L, Baker S (1996). Sonographic appearance of the endometrium and ovaries during cycles stimulated with human menopausal gonadotropin.. J Reprod Med..

[30] Bergh C, Hillensjo T, Nilsson L (1992). Sonographic evaluation of the endometrium in in vitro fertilization IVF cycles. A way to predict pregnancy?. Acta Obstet Gynecol Scand..

[31] Isaacs JD, Jr, Wells CS, Williams DB, Odem RR, Gast MJ, Strickler RC (1996). Endometrial thickness is a valid monitoring parameter in cycles of ovulation induction with menotropins alone.. Fertil Steril..

[32] Oliveira JB, Baruffi RL, Mauri AL, Petersen CG, Borges MC, Franco JG, Jr (1997). Endometrial ultrasonography as a predictor of pregnancy in an in-vitro fertilization programme after ovarian stimulation and gonadotrophin-releasing hormone and gonadotrophins.. Hum Reprod..

[33] Sundstrom P (1998). Establishment of a successful pregnancy following in-vitro fertilization with an endometrial thickness of no more than 4 mm.. Hum Reprod..

[34] Quintero RB, Sharara FI, Milki AA (2004). Successful pregnancies in the setting of exaggerated endometrial thickness.. Fertil Steril..

[35] Azumaguchi A, Henmi H, Saito M, Itabashi M (2011). Role of dilatation and curettage in the etiology of thin endometrium.. Abstracts of the 27th Annual Meeting of ESHRE..

[36] Better health channel. Dilatation and curettage, fact sheet.. http://www.betterhealth.vic.gov.au..

[37] Mansur MM (1992). Ultrasound diagnosis of complete abortion can reduce need for curettage.. Eur J Obstet Gynecol Reprod Biol..

[38] (2008). X-Plain, Dilatation and Curettage: Reference summary.. Educational sheet..

[39] Goldstein SR (2009). The role of transvaginal ultrasound or endometrial biopsy in the evaluation of the menopausal endometrium.. Am J Obstet Gynecol..

[40] Bettocchi S, Ceci O, Vicino M, Marello F, Impedovo L, Selvaggi L (2001). Diagnostic inadequacy of dilatation and curettage.. Fertil Steril..

[41] Henshaw RC, Templeton AA (1993). Methods used in first trimester abortion.. Current obstetrics and gynecology..

[42] MacKenzie IZ (1992). Endometrial biopsy.. Current obstetrics and gynecology..

[43] Polishuk W (1975). Endometrial regeneration and adhesion formation.. African J Obst and Gyn..

[44] Montgomery BE, Daum GS, Dunton CJ (2004). Endometrial hyperplasia: a review.. Obstet Gynecol Surv..

[45] Deckardt R, Lueken RP, Gallinat A, Moller CP, Busche D, Nugent W (2002). Comparison of transvaginal ultrasound, hysteroscopy, and dilatation and curettage in the diagnosis of abnormal vaginal bleeding and intrauterine pathology in perimenopausal and postmenopausal women.. J Am Assoc Gynecol Laparosc..

[46] Moon KS, Richter KS, Levy MJ, Widra EA (2009). Does dilation and curettage versus expectant management for spontaneous abortion in patients undergoing in vitro fertilization affect subsequent endometrial development?. Fertil Steril..

[47] Epstein E, Ramirez A, Skoog L, Valentin L (2001). Dilatation and curettage fails to detect most focal lesions in the uterine cavity in women with postmenopausal bleeding.. Acta Obstet Gynecol Scand..

[48] Yuval Y, Lipitz S, Dor J, Achiron R (1999). The relationships between endometrial thickness, and blood flow and pregnancy rates in in-vitro fertilization.. Hum Reprod..

[49] Li L, Shi J, Zhang QF, Yan J, Yan LY, Shen F (2011). Effect of curettage and copper wire on rabbit endometrium: a novel rabbit model of endometrial mechanical injury.. Chin Med J (Engl)..

